# Supplementary material for: Feasibility of Predicting Surgical Duration in Endometriosis Using Numerical Multi-Scoring System of Endometriosis (NMS-E)
Source: Biomedicines. 2024 Jun 6;12(6):1267. doi: 10.3390/biomedicines12061267 (PMC11201286; doi:10.3390/biomedicines12061267)
Supplement: Supplementary file 1 [file biomedicines-12-01267-s001.zip › Supplementary NMS-E format PP.ver.4.pptx]

## Slide 1
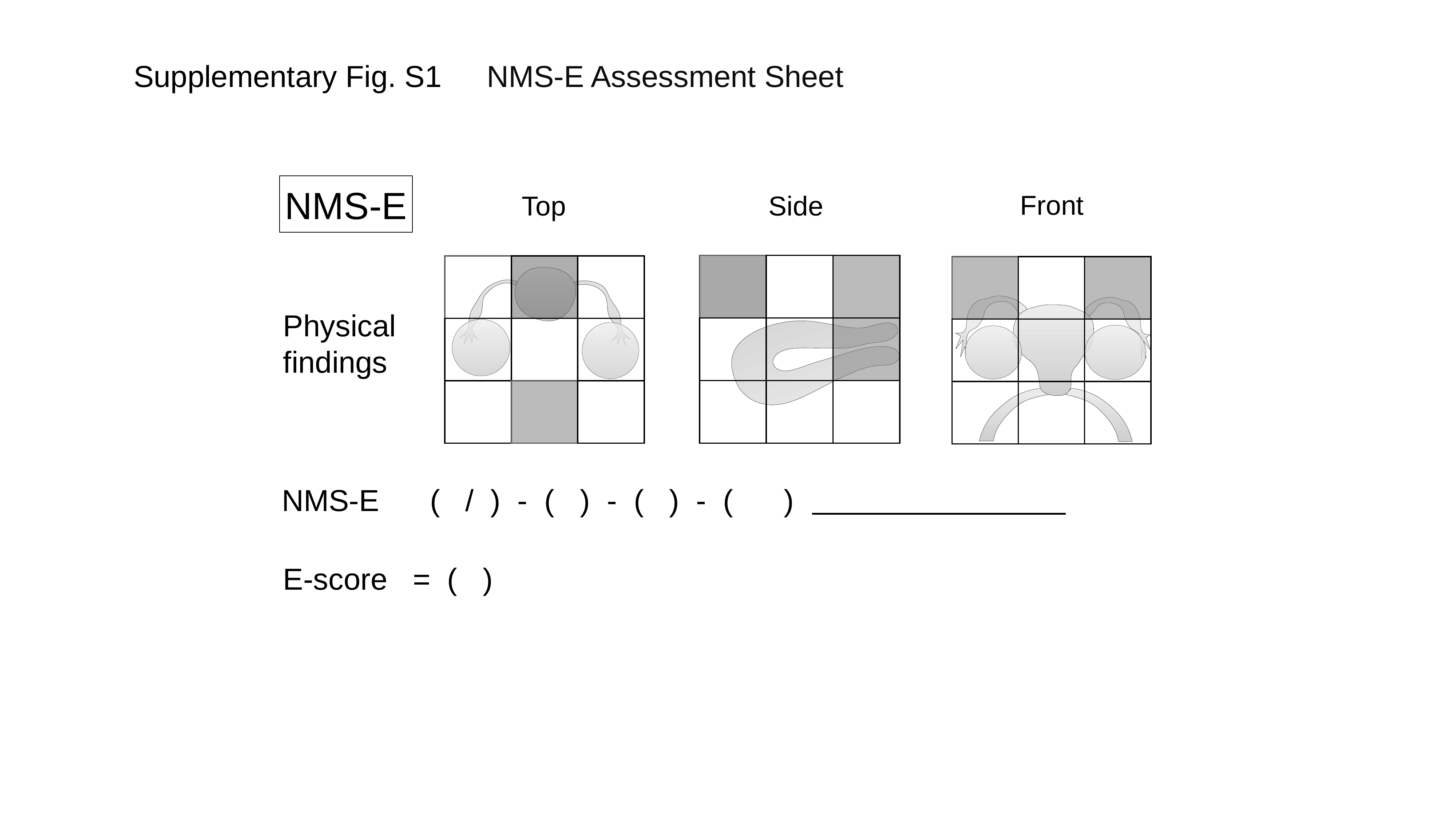

Supplementary Fig. S1　NMS-E Assessment Sheet
NMS-E
Front
Side
Top
| | | |
| --- | --- | --- |
| | | |
| | | |
| | | |
| --- | --- | --- |
| | | |
| | | |
| | | |
| --- | --- | --- |
| | | |
| | | |
Physical
findings
NMS-E ( / ) - ( ) - ( ) - ( )
E-score = ( )

## Slide 2
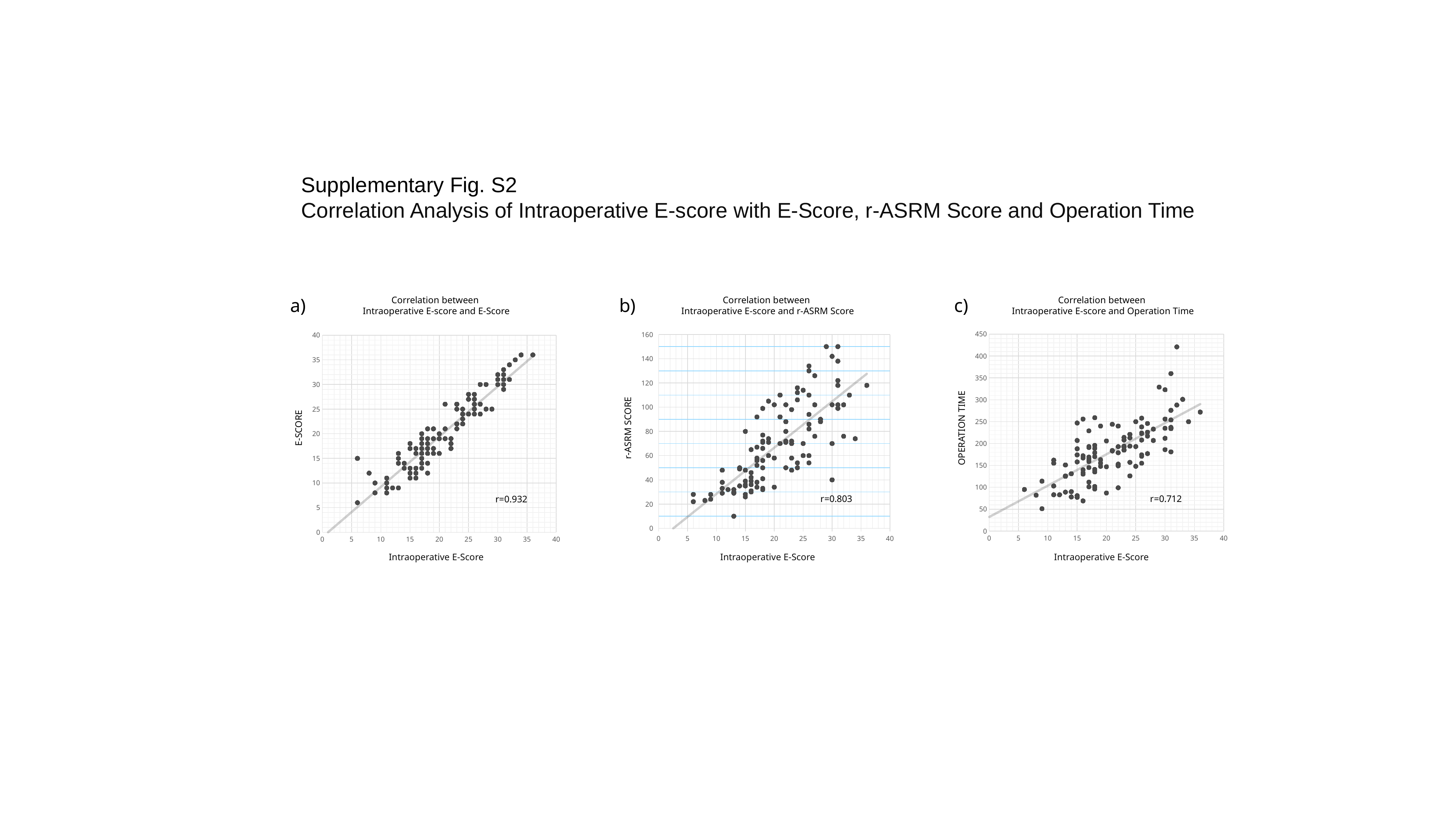

Supplementary Fig. S2
Correlation Analysis of Intraoperative E-score with E-Score, r-ASRM Score and Operation Time
b)
c)
a)
Correlation between
Intraoperative E-score and E-Score
Correlation between
Intraoperative E-score and r-ASRM Score
Correlation between
Intraoperative E-score and Operation Time
### Chart
| Category | |
|---|---|
### Chart
| Category | |
|---|---|
### Chart
| Category | |
|---|---|OPERATION TIME
E-SCORE
r-ASRM SCORE
r=0.932
Intraoperative E-Score
Intraoperative E-Score
Intraoperative E-Score

## Slide 3
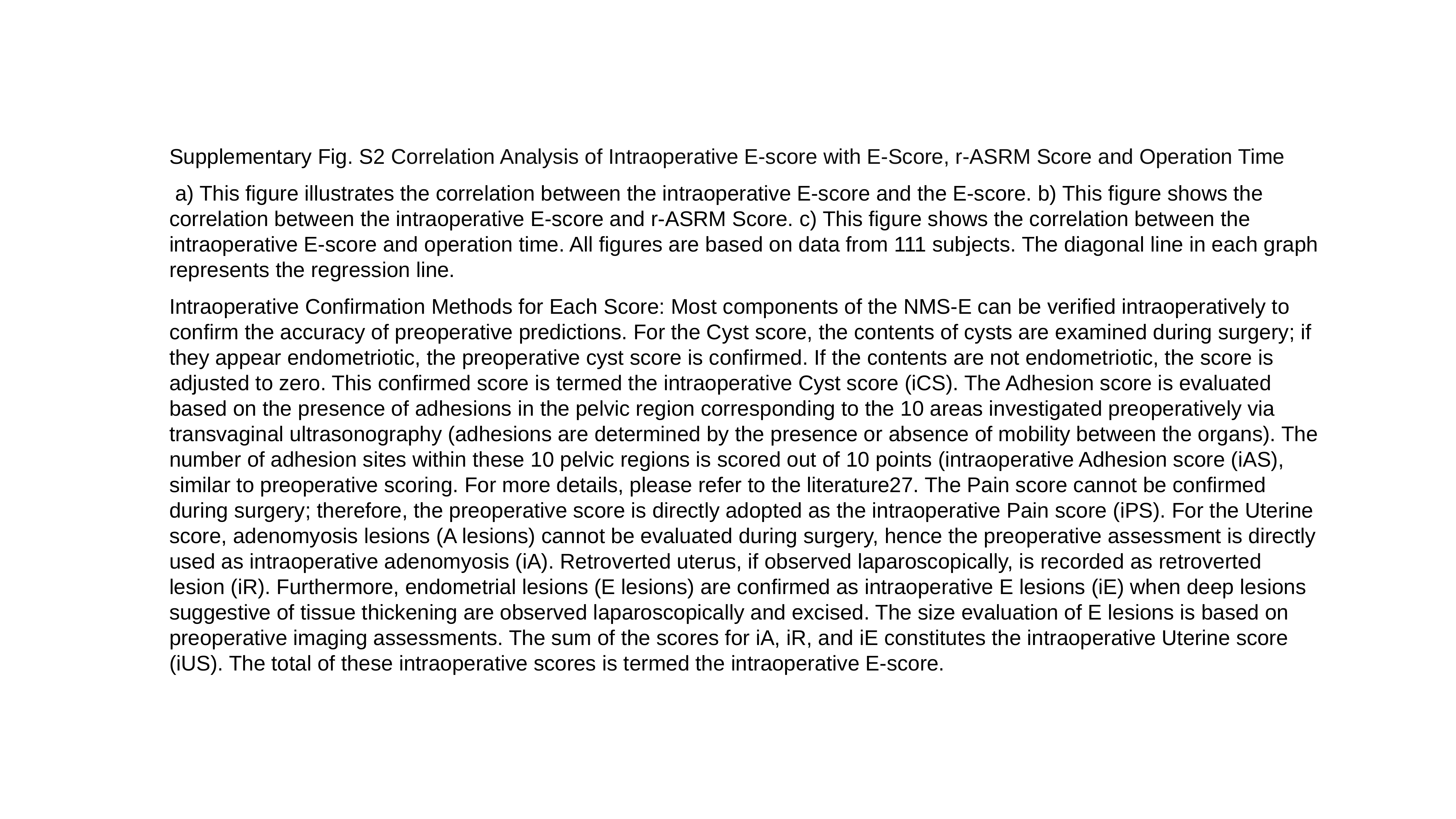

Supplementary Fig. S2 Correlation Analysis of Intraoperative E-score with E-Score, r-ASRM Score and Operation Time
 a) This figure illustrates the correlation between the intraoperative E-score and the E-score. b) This figure shows the correlation between the intraoperative E-score and r-ASRM Score. c) This figure shows the correlation between the intraoperative E-score and operation time. All figures are based on data from 111 subjects. The diagonal line in each graph represents the regression line.
Intraoperative Confirmation Methods for Each Score: Most components of the NMS-E can be verified intraoperatively to confirm the accuracy of preoperative predictions. For the Cyst score, the contents of cysts are examined during surgery; if they appear endometriotic, the preoperative cyst score is confirmed. If the contents are not endometriotic, the score is adjusted to zero. This confirmed score is termed the intraoperative Cyst score (iCS). The Adhesion score is evaluated based on the presence of adhesions in the pelvic region corresponding to the 10 areas investigated preoperatively via transvaginal ultrasonography (adhesions are determined by the presence or absence of mobility between the organs). The number of adhesion sites within these 10 pelvic regions is scored out of 10 points (intraoperative Adhesion score (iAS), similar to preoperative scoring. For more details, please refer to the literature27. The Pain score cannot be confirmed during surgery; therefore, the preoperative score is directly adopted as the intraoperative Pain score (iPS). For the Uterine score, adenomyosis lesions (A lesions) cannot be evaluated during surgery, hence the preoperative assessment is directly used as intraoperative adenomyosis (iA). Retroverted uterus, if observed laparoscopically, is recorded as retroverted lesion (iR). Furthermore, endometrial lesions (E lesions) are confirmed as intraoperative E lesions (iE) when deep lesions suggestive of tissue thickening are observed laparoscopically and excised. The size evaluation of E lesions is based on preoperative imaging assessments. The sum of the scores for iA, iR, and iE constitutes the intraoperative Uterine score (iUS). The total of these intraoperative scores is termed the intraoperative E-score.
